# Aromatic L-amino acid decarboxylase deficiency in countries in the Middle East: a case series and literature review

**DOI:** 10.1007/s00431-023-04886-5

**Published:** 2023-03-16

**Authors:** Musaad Abukhaled, Mohammed Al Muqbil, Malak Ali Alghamdi, Khalid Hundallah, Jehan Suleiman, Tawfeg Ben-Omran, Majid Alfadhel, Mohammed Almannai, Rehab Alsaleh, Brahim Tabarki

**Affiliations:** 1 Department of Neurosciences, King Faisal Specialist Hospital and Research Center, (KFSH-RC), Riyadh, Saudi Arabia; 2 College of Medicine, King Saud bin Abdulaziz University for Health Sciences (KSAU-HS), Riyadh, Saudi Arabia; 3 Division of Pediatric Neurology, Department of Pediatrics, King Abdullah Specialized Children’s Hospital, National Guard Health Affairs (NGHA), Riyadh, Saudi Arabia; 4 King Abdullah International Medical Research Center, Ministry of National Guard Health Affairs (MNG-HA), Riyadh, Saudi Arabia; 5 Medical Genetic Division, Pediatric Department, College of Medicine, King Saud University, Riyadh, Saudi Arabia; 6 Division of Neurology, Department of Pediatrics, Prince Sultan Military Medical City, Riyadh, Saudi Arabia; 7 Division of Neurology, Department of Pediatrics, Tawam Hospital, Al Ain, United Arab Emirates; 8 College of Medicine and Health Sciences, United Arab Emirates University, Al Ain, United Arab Emirates; 9 American Center for Psychiatry and Neurology, Abu Dhabi, United Arab Emirates; 10 Sidra Medicine and Research Center, Doha, Qatar; 11 Hamad Medical Corporation, Doha, Qatar; 12 Department of Genetics and Precision Medicine, Department of Pediatrics, King Saud bin Abdulaziz University for Health Sciences, King Abdulaziz Medical City, Riyadh, Saudi Arabia; 13 Medical Genomic Research Department, King Abdullah International Medical Research Centre (KAIMRC), King Saud bin Abdulaziz University for Health Sciences (KSAU-HS), Ministry of National Guard Health Affairs (MNG-HA), Riyadh, Saudi Arabia

**Keywords:** AADC deficiency, Delayed diagnosis, Developmental delay, Whole exome sequencing, Case report

## Abstract

Aromatic L-amino acid decarboxylase (AADC) deficiency is a rare inherited neurometabolic disorder that can lead to severe physical and developmental impairment. This report includes 16 patients from the Middle East and is the largest series of patients with confirmed AADC deficiency from this region reported to date. The patients displayed a range of signs and symptoms at presentation and almost all failed to reach major motor milestones. Missed and delayed diagnoses were common leading to the late introduction of targeted treatments. Eight unique variants were identified in the *DDC* gene, including six missense and two intronic variants. A previously undescribed variant was identified: an intronic variant between exons 13 and 14 (c.1243-10A>G). The patients were mostly treated with currently recommended medications, including dopamine agonists, vitamin B6, and monoamine oxidase inhibitors. One patient responded well, but treatment outcomes were otherwise mostly limited to mild symptomatic improvements. Five patients had died by the time of data collection, confirming that the condition is associated with premature mortality. There is an urgent need for earlier diagnosis, particularly given the potential for gene therapy as a transformative treatment for AADC deficiency when provided at an early age.

*  Conclusions*: Delays in the diagnosis of AADC deficiency are common. There is an urgent need for earlier diagnosis, particularly given the potential for gene therapy as a transformative treatment for AADC deficiency when provided at an early age.

**Table:** 

**What is Known:** *• Aromatic L-amino acid decarboxylase deficiency is a rare neurometabolic disorder that can lead to severe physical and developmental impairment.* *• Currently recommended medications provide mostly mild symptomatic improvements.*	
**What is New:** *• The clinical presentation of sixteen patients with confirmed AADC deficiency varied considerably and almost all failed to reach major motor milestones.* *• There is an urgent need for earlier diagnosis, given the potential for gene therapy as a transformative treatment for AADC deficiency when provided at an early age.*

**What is Known:**

*• Aromatic L-amino acid decarboxylase deficiency is a rare neurometabolic disorder that can lead to severe physical and developmental impairment.*

*• Currently recommended medications provide mostly mild symptomatic improvements.*

**What is New:**

*• The clinical presentation of sixteen patients with confirmed AADC deficiency varied considerably and almost all failed to reach major motor milestones.*

*• There is an urgent need for earlier diagnosis, given the potential for gene therapy as a transformative treatment for AADC deficiency when provided at an early age.*

## Introduction

First described in 1990, aromatic L-amino acid decarboxylase (AADC) deficiency is an ultrarare, autosomal recessive, neurotransmitter metabolic disorder resulting from pathogenic variants within the dopa decarboxylase (*DDC*) gene [1, 2]. To date, at least 261 cases have been reported in the medical literature [3] and, as of June 2022, there are currently 420 variants listed in the Pediatric Neurotransmitter Disease database (PNDdb; available at: http://biopku.org/pnddb/home.asp), including 370 that are associated with a neurotransmitter deficiency phenotype. Although the global prevalence of AADC deficiency is unknown, it is believed to be higher in Asian populations owing to the presence of the founder variant c.714+4A>T [4]. In Taiwan, a pilot newborn screening project indicated that the incidence of AADC deficiency was 1:32,000 [5], whereas in the USA, Europe, and Japan, birth rates of 1:42:000–1:90,000, 1:116,000, and 1:162,000, respectively, were estimated [6–8].

The AADC enzyme is required for the final step in the synthesis of monoamine neurotransmitters. Deficiency of this enzyme results in a loss of dopamine, serotonin, epinephrine, and norepinephrine production leading to severe physical and intellectual disabilities and risk of early death [1, 9, 10]. The symptom spectrum can be highly variable including motor delay, hypotonia, dystonia, oculogyric crises, autonomic symptoms, and behavioral problems [10]. Most reported cases have no or very limited attainment of motor or developmental milestones [9].

There is a significant heterogeneous phenotypic spectrum and symptoms can be non-specific, and the diagnosis of AADC deficiency is frequently delayed, with patients often misdiagnosed with other more common conditions, such as cerebral palsy or seizure disorders [11]. Lack of awareness of this rare condition among primary care physicians may also mean that clinical suspicion of this disease is low, further contributing to diagnostic delays [12]. Key diagnostic tests for AADC deficiency include the analysis of neurotransmitter metabolites in cerebrospinal fluid (CSF), measurement of AADC activity in plasma, and genetic testing to identify pathogenic variants in the *DDC* gene. Routine brain magnetic resonance imaging (MRI) is not required for the diagnosis of AADC deficiency as no specific pattern of changes has been observed; however, it can be beneficial for ruling out cerebral palsy as a differential diagnosis. Similarly, electroencephalography (EEG) is not required for diagnosis, although it can be useful to differentiate oculogyric crises from epileptic events [9]. AADC deficiency can also be identified by measurement of 3-O-methyldopa (3-OMD) in dried blood spots, providing the potential for newborn screening for this disease [5].

Currently recommended first-line pharmacological treatments include dopamine agonists, monoamine oxidase (MAO) inhibitors, and pyridoxine/pyridoxal phosphate (vitamin B6). However, treatment response is often disappointing, offering, at best, mild symptomatic improvements and do not treat the underlying enzyme deficiency [9]. Gene therapy will expand treatment options in the future, with recent studies demonstrating that replacement with a functional copy of the *DDC* gene in the basal ganglia region of the brain using an adeno-associated viral vector leads to clinical improvements [10, 13–15]. These improvements correlated with younger age in gene therapy trials, indicating that there is a need for earlier diagnosis of AADC deficiency [13, 14].

The objective of this study was to describe the clinical presentation and diagnostic workup for 16 patients with confirmed AADC deficiency from the Gulf Cooperation Council countries; the largest number of AADC deficiency patients included in a single case series from the Middle East.

## Materials and methods

Sixteen patients with confirmed AADC deficiency were included in this case series. Clinical, laboratory, and treatment data were retrospectively collected from patients’ electronic medical records. Consent for inclusion in this study was provided by all patients and/or their families.

## Results

### Patient demographics

Patient demographics for this case series are shown in Table 1. Sixteen patients were included, six females and ten males. Age at diagnosis of AADC deficiency ranged from 10 months to 18 years. All patients had consanguineous parents and the majority of patients failed to reach motor or developmental milestones. Two patients were able to walk (patient 2 and patient 11). Five patients had a positive family history of AADC deficiency, with three patients having at least one affected sibling and two patients having one affected first cousin. A further four patients had a potentially relevant family history; two had a sibling diagnosed with cerebral palsy (deceased), one patient had a cousin with developmental delay, and one had parents who had previously experienced recurrent, unexplained abortions. The remaining seven patients had no relevant family history. Five patients died prior to data collection. Patients’ age at death was confirmed as 3, 9, and 10 years of age for the three patients with available data (Table 1).

**Table 1:** Patient demographics

**Patient**	**Sex**	**Consanguinity**	**Relevant family history**	**Motor and cognitive function**	**Vital status** **(age as of 1 June 2022)**
1	F	Y	1 sibling (deceased) diagnosed with CP	Bedridden, verbal function: no words	Alive (8 years of age)
2	M	Y	No	Normal motor, speech and cognitive function	Alive (8 years of age)
3	M	Y	No	Bedridden, verbal function: no words	Alive (5 years of age)
4	F	Y	Recurrent, unexplained abortions	Bedridden, verbal function: no words	Alive (6 years of age)
5	M	Y	No	NA^e^	Alive (4 years of age)
6	M	Y	Recurrent, unexplained abortions. **1 cousin with AADCd**^**a**^**;** 1 sibling (deceased) and 1 cousin with suspected CP	NA^e^	Deceased (Age at death: 10 years of age)
7^b^	M	Y	No	NA^e^	Deceased (Age at death: 9 years of age)
8^c^	F	Y	1 sibling (deceased) diagnosed with CP, 1 sibling with similar symptom presentation (AADCd diagnosis not confirmed)	Bedridden, verbal function: no words	Deceased (Age at death: 3 years of age)
9^c^	F	Y	2 siblings with similar symptom presentation (AADCd diagnosis not confirmed); **1 sibling known to be a carrier**	Bedridden, verbal function: no words	Alive (21 years of age as of March 2022)
10^c^	M	Y	1 cousin with developmental delay (AADCd diagnosis not confirmed)	Bedridden, verbal function: no words	Alive (5 years of age)
11	M	Y	**1 sibling with AADCd**^**d**^	NA^e^	Alive (7 years of age)
12	F	Y	**1 sibling with AADCd**^**d**^	NA^e^	Alive (16 years of age)
13	F	Y	No	NA^e^	Alive (2 years of age)
14	M	Y	No	NA^e^	Alive (10 months of age)
15	M	Y	No	NA^e^	Deceased
16	M	Y	**1 cousin diagnosed with AADCd**^**a**^	NA^e^	Deceased

Demographics and characteristics of the 16 patients included in this case series

*AADCd* aromatic L-amino acid decarboxylase deficiency, *CP* cerebral palsy, *F* female, *M* male, *NA* not available, *Y* yes

^a^Patient 6 and 16 are cousins

^b^Patient 7 has previously been published in a case report [31]

^c^Patients 8, 9, and 10 have previously been published in a case report [30]

^d^Patients 11 and 12 are siblings

^e^Information not provided by the author

### Presenting signs and symptoms

**Fig. 1:**
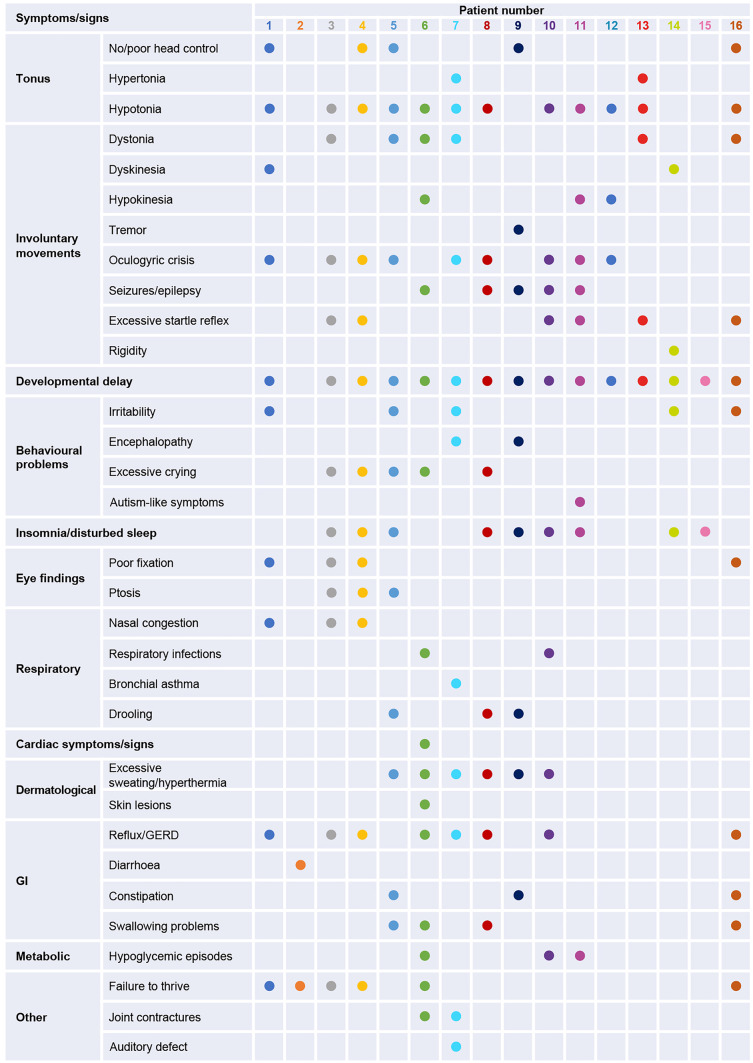
Symptoms and signs reported in the 16 AADCd patient cases. Symptoms and signs at presentation. GERD, gastroesophageal reflux disease; GI, gastrointestinal

**Fig. 2:**
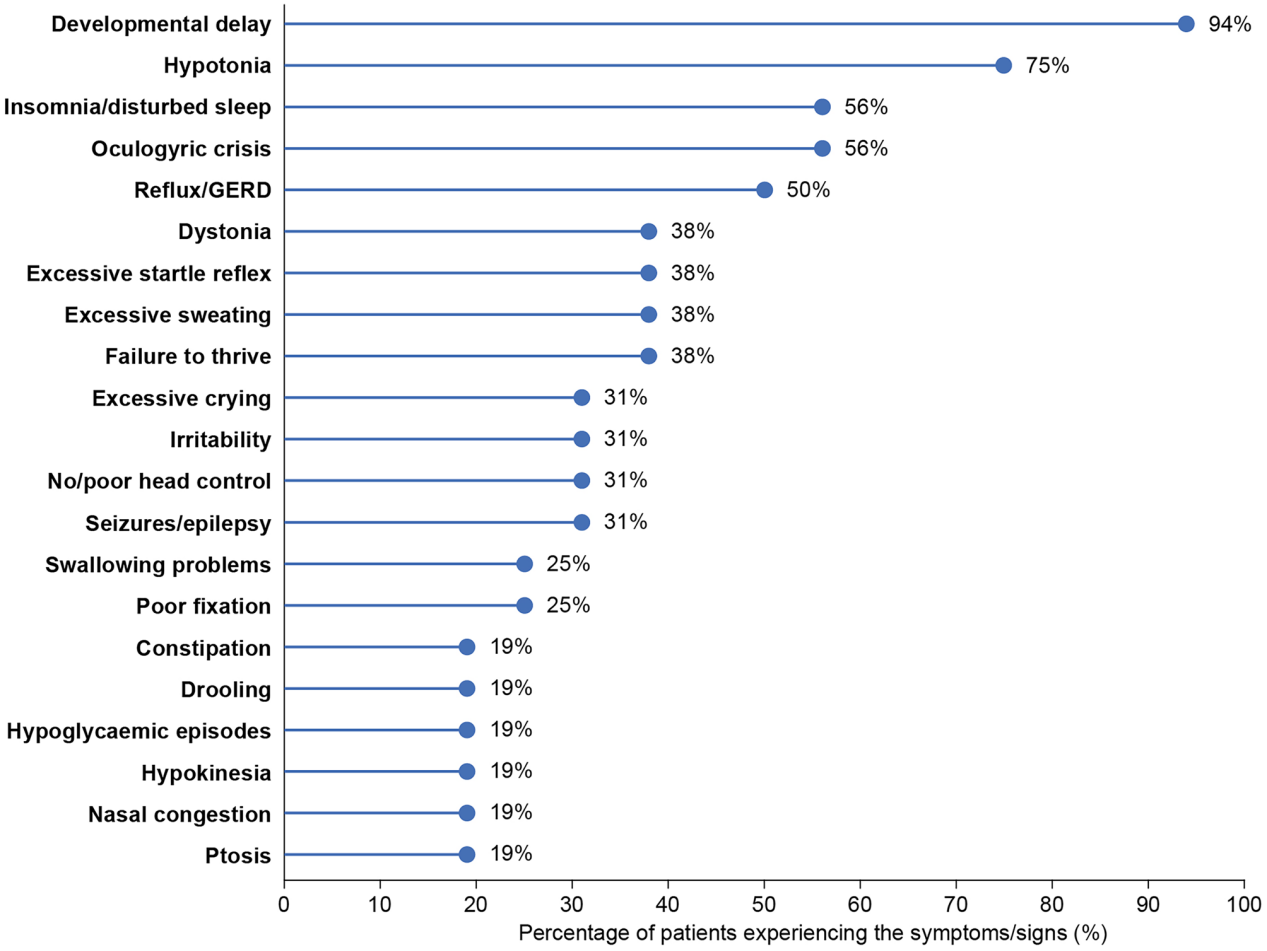
Prevalence of symptoms and signs. Symptoms and signs experienced by at least 3 patients at presentation. GERD, gastroesophageal reflux disease

Most of the patients included in this case series presented with symptoms of AADC deficiency prior to 12 months of age. The range and number of symptoms and signs were highly variable between patients (Fig. 1), with the most commonly reported symptoms being developmental delay (94%), hypotonia (75%), and insomnia/disturbed sleep and oculogyric crises (both 56%) (Fig. 2). Most patients had symptoms related to muscle tone (*n*=13), including hypotonia, no or poor head control, and hypertonia. In addition, the majority of the patients experienced gastrointestinal symptoms (*n*=11), including reflux/gastroesophageal reflux disease (GERD), diarrhea, constipation, and feeding/swallowing problems. Failure to thrive was also common (*n*=6).

### Diagnosis

**Fig. 3:**
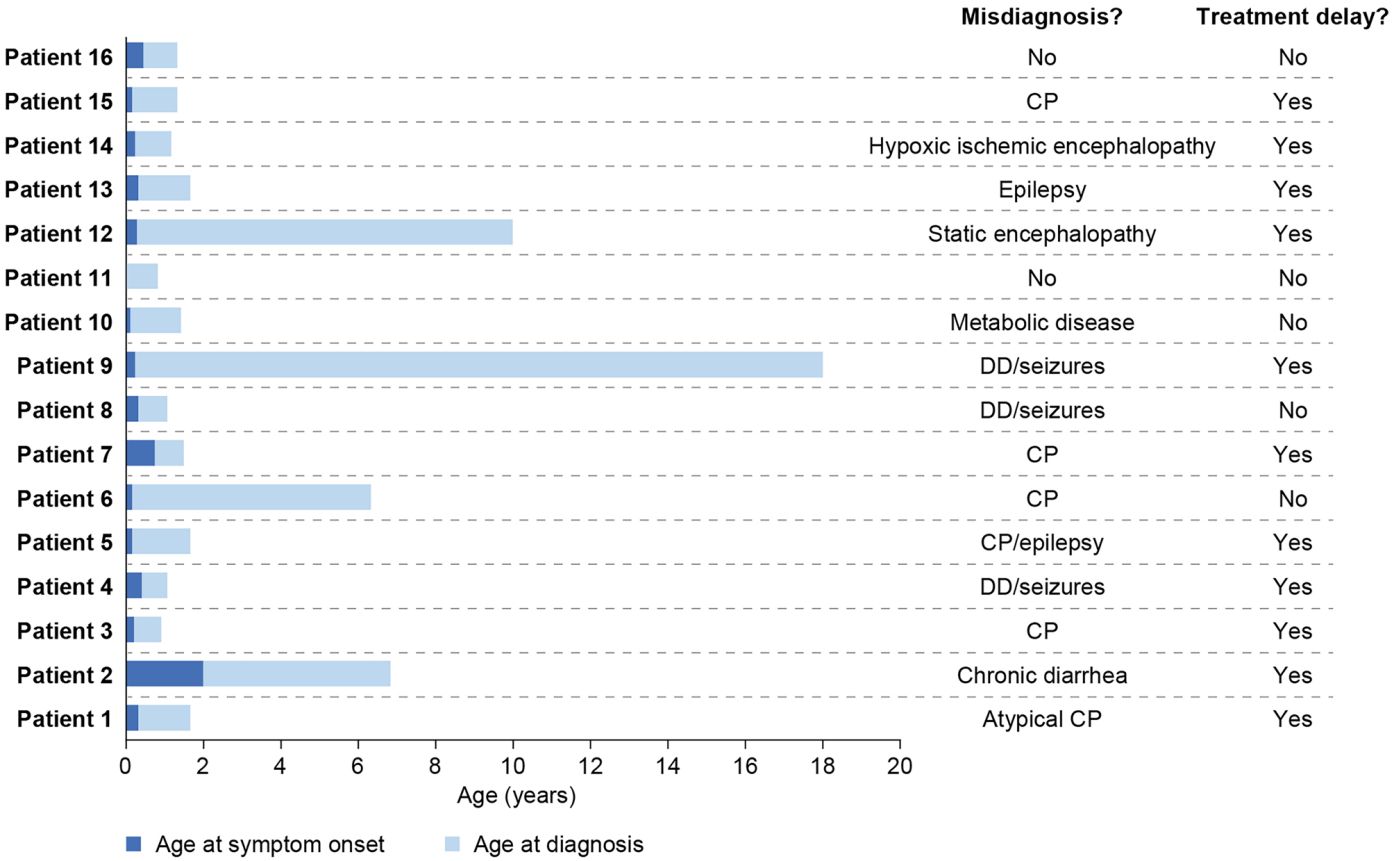
Symptom onset and diagnosis. Age at symptom onset and diagnosis. CP, cerebral palsy; DD, developmental delay

Although most patients had severe motor dysfunction and developmental delay, diagnosis was generally delayed, with only seven patients receiving a diagnosis within 1 year of symptom onset (Fig. 3). For four patients, diagnosis was markedly delayed: occurring 5, 6, 10, and 18 years after symptom onset for patients 2, 6, 12, and 9, respectively. Most patients (*n*=14) had been misdiagnosed prior to receiving a diagnosis of AADC deficiency, with cerebral palsy and seizures being common misdiagnoses. The two patients who did not receive a misdiagnosis had a positive family history for AADC deficiency. For many patients (*n*=11), the misdiagnosis resulted in delayed access to targeted treatments for AADC deficiency.

As part of the diagnostic workup, patients underwent a range of tests, including brain MRI (*n*=15), EEG (*n*=13), and biochemical analyses (*n*=10) (Table 2). Abnormal MRI findings included signs of brain atrophy in three patients and minor abnormalities that may represent metabolic derangement in one patient. Some minor EEG changes were reported for five patients, but no epileptic activity was seen. In most cases, MRI and EEG results were normal or there were no specific findings. Six patients had a CSF analysis performed, with all patients displaying a profile characteristic of AADC deficiency, i.e., low levels of 5-hydroxyindoleacetic acid and homovanillic acid and elevated 3-OMD, L-dopa, and 5-hydroxytryptophan. Seven patients had plasma AADC enzyme activity measured, all of which were significantly lower than normal (reference value 47–119 pmol/mL/min [16]) and close to the detection limit of the assay. Only one patient had dried blood spot 3-OMD measured, which was elevated, indicative of AADC deficiency.

**Table 2:** Diagnostic tests

**Patient**	**Brain MRI**	**EEG**	**CSF neurotransmitter concentrations (nmol/L)**					**Plasma AADC activity (pmol/mL/min)**	**Dried blood spot 3-OMD (nmol/L)**
			**5-HIAA**	**HVA**	**3-OMD**	**L-Dopa**	**5-HTP**		
**1**	Normal	Normal	**91**	**65**	**2000**	ND	ND	**6**	ND
**2**	ND	ND	ND	ND	ND	ND	ND	**10**	**2060**
**3**	**Non-specific brain atrophy**	Normal	ND	ND	ND	ND	ND	**< 5**	ND
**4**	Normal	Normal	**19**	**33**	ND	ND	170	**< 5**	ND
**5**	Normal	Normal	ND	ND	ND	ND	ND	**2.5**^a^	ND
**6**	Normal	Normal	**21**	**13**	**943**	ND	ND	ND	ND
**7**	Normal	Severe background depression	**93**	**60**	**2076**	**91**	**274**	**4**	ND
**8**	Normal	Intermittent slow activity, bilateral mid-posterior temporal and occipital	**6**	**38**	**> 2500**	ND	ND	ND	ND
**9**	Normal	Mild-to-moderate diffuse encephalopathy	ND	ND	ND	ND	ND	ND	ND
**10**	**Minor abnormalities**^b^	Non-specific bioccipital abnormality	**7**	**20**	**1171**	ND	ND	ND	ND
**11**	Normal	Normal apart from slow background	ND	ND	ND	ND	ND	ND	ND
**12**	Normal	Normal	ND	ND	ND	ND	ND	**0.65**	ND
**13**	Normal	Normal	ND	ND	ND	ND	ND	ND	ND
**14**	**Atrophy**	Normal	ND	ND	ND	ND	ND	ND	ND
**15**	**Atrophy**	ND	ND	ND	ND	ND	ND	ND	ND
**16**	Normal	NA^c^	ND	ND	ND	ND	ND	ND	ND

Results of diagnostic tests. Abnormal findings/values (outside of reference ranges provided for the assays used) are indicated in bold

*5-HIAA* 5-hydroxyindoleactetic acid, *5-HTP* 5-hydroxytryptophan, *AADC* aromatic L-amino acid decarboxylase, *CSF* cerebrospinal fluid, *EEG* electroencephalogram, *HVA* homovanillic acid, *MRI* magnetic resonance imaging, *NA* not available, *ND* not done, *OMD* O-methyldopa

^a^Measured indirectly by dopamine level

^b^Tiny non-specific foci of signal abnormality, which might represent minor markers of metabolic derangement

^c^Information not provided by the author

**Fig. 4:**
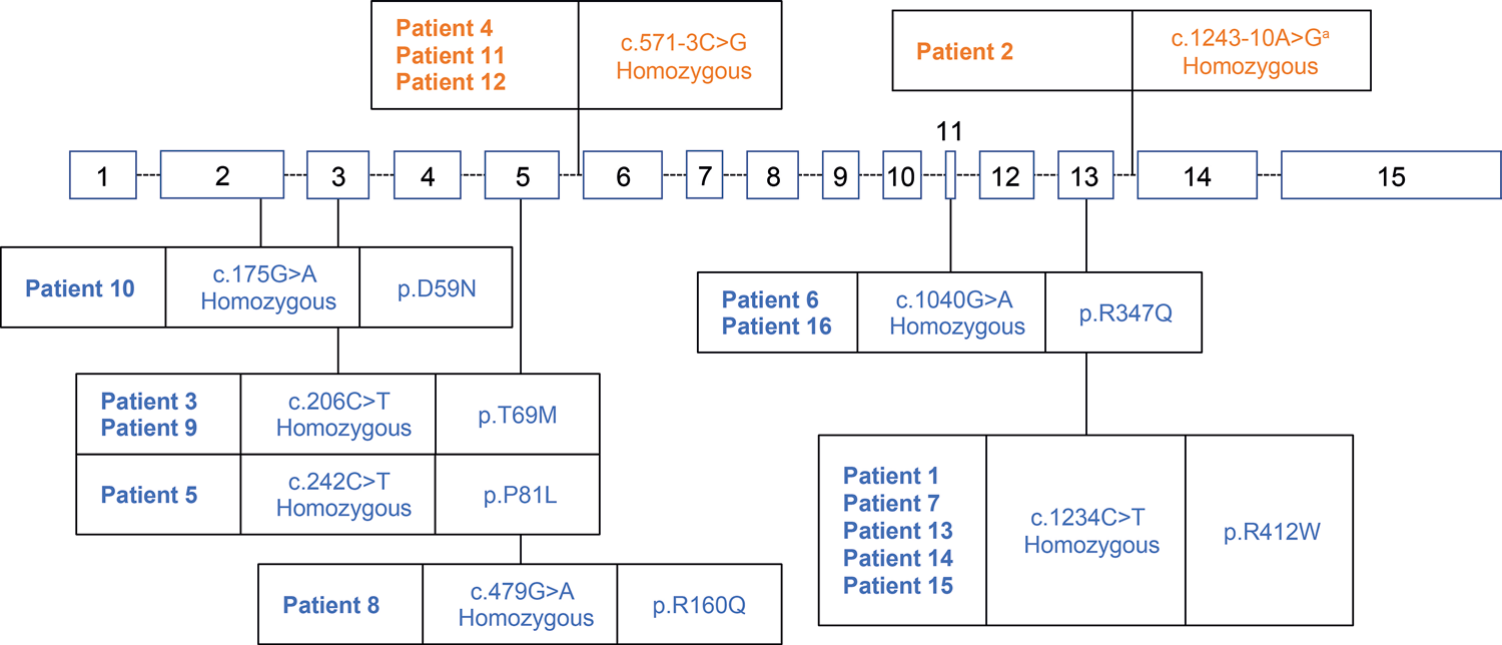
*DDC* gene variants. Genomic organization of the *DDC* gene and location of variants. Missense variants are indicated in blue and intron variants are indicated in orange. ^a^Previously unpublished variant. *DDC*, dopa decarboxylase

All patients had a full *DDC* gene sequence performed. Eight unique variants were identified, of which one intronic variant was previously unpublished (Fig. 4). Six missense variants leading to a single amino acid substitution were reported for 12 patients across exons 2 (*n*=1), 3 (*n*=3), 5 (*n*=1), 11 (*n*=2) and 13 (*n*=5). Two intronic variants affecting four patients were reported. All patients had homozygous variants.

### Treatment and outcomes

Many of the patients were treated with the recommended first-line pharmacological treatments, i.e., dopamine agonists (*n*=9), vitamin B6 (*n*=13), and MAO inhibitors (*n*=3) (Table 3). A further four patients received folinic acid and three received levodopa/carbidopa. Symptomatic treatments included benzodiazepines, anti-epileptics, anti-cholinergic drugs, and α_2_-adrenergic agonists.

**Table 3:** Therapeutic interventions

**Patient**	**AADC deficiency treatment**	**Treatment response**
1	• Bromocriptine 10 mg/day • Vitamin B6 100 mg/day	Mild improvements in oculogyric crises
2	• Vitamin B6 200 mg/day	Moderate improvements in diarrhea
3	• Bromocriptine 7.5 mg/day • Vitamin B6 100 mg/day • Clonazepam 0.1 mg/kg/day	Mild improvements in head control and oculogyric crises
4	• Bromocriptine 15 mg/day • Vitamin B6 200 mg/day • Clonazepam 0.1 mg/kg/day	Mild improvement in motor control and oculogyric crisis
5	• Bromocriptine 5.5 mg/day • Vitamin B6 100 mg/day • Selegiline 4.5 mg/day • Trihexyphenidyl 12 mg/day • Clonidine 0.1 mg/day	Improved head control, improvement in dystonic and oculogyric crises
6	• Vitamin B6 – dose details unavailable • Pramipexole – dose details unavailable	NA^a^
7	• Bromocriptine 20 mg/day • Vitamin B6 200 mg/day	No symptomatic improvements following initiation of treatment. Medication stopped after 2 months
8	• Vitamin B6 100 mg morning, 200 mg night • Folinic acid 5 mg/day • Levetiracetam 150 mg/day • Carbidopa/levodopa 5 mg/day	No
9	• Clonazepam 0.5 mg morning, 1 mg evening • Levetiracetam 2000 mg/day	No
10	• Pramipexole 0.375 mg/day • Vitamin B6 100 mg/day	Improvement in seizures
11	• Bromocriptine 12 mg/day • Benztropine 0.1 mg/kg/day • Folinic acid 1 mg/day • Selegiline 1.2 mg/day	Patient is able to walk, speak, and attend school
12	• Bromocriptine 12 mg/day • Vitamin B6 200 mg/day • Benztropine 0.04–0.1 mg/kg/day • Folinic acid 1 mg/day • Selegiline 1.25 mg/day	Better movement and tone, less drooling. Some improvement in oculogyric crises
13	• Vitamin B6 200 mg/day • Folinic acid 2 mg/kg/day • Trihexyphenidyl 5 mg morning, 10 mg evening	No
14	• Carbidopa/levodopa 7.8 mg/kg/day Vitamin B6 80 mg/day	No
15	• Carbidopa/levodopa 12 mg/kg/day • Vitamin B6 80 mg/day	Poor response
16	• NA^a^	NA^a^

Medical treatments and response to treatment. Patient 9 was transferred from another center at age 15 and had previously been treated with levetiracetam, phenytoin and lacosamide; patient 10 was previously treated with carbamazepine (15 mg/kg/day) and levetiracetam (50 mg/kg/day); however, treatments were changed after a consultation in the USA, during which the patient was told to stop all anti-epileptic drugs; patient 10 required dose adjustment with pramipexole owing to experience of abnormal movement with dose increase

*AADC* aromatic L-amino acid decarboxylase, *NA* not available

^a^Information not provided by the author

Six patients were judged to have not responded to treatment or to have had a poor response. Five patients exhibited mild-to-moderate improvements in symptoms, including oculogyric crises, and one patient exhibited improvements in dystonic and oculogyric crises. Patient 11 received early treatment with bromocriptine, benztropine, folinic acid, and selegiline and had a very good response. He is currently able to walk, speak, and attend school. Conversely, his sibling (patient 12) with the same genotype commenced treatment later and did not have such a good response, although some improvements in movement and tone were noted. Five patients were deceased at the time of data collection (Table 1).

## Discussion

This report describes the clinical characteristics, diagnostic workup, and treatment of 16 patients with AADC deficiency managed in specialist centers within the Middle East.

At a young age, the majority of patients presented with severe motor dysfunction and had no or very limited attainment of developmental milestones. As has been previously reported, sex was not found to be associated with phenotype [9]. Signs and symptoms were consistent with other case reports for AADC deficiency [17, 18], with motor issues, global developmental delay, and gastrointestinal symptoms reported frequently. The specific pattern of signs and symptoms varied considerably across the 16 cases, which is also consistent with other case series [17–19]. Of note, oculogyric crises have previously been described as a near-universal feature of AADC deficiency [17–19]; however, in our case series, only 9 out of 16 patients experienced this symptom. As one of the more specific symptoms for AADC deficiency, oculogyric crises can help spark clinical suspicion for the disease. This case series highlights that lack of oculogyric crises should not exclude patients from screening for AADC deficiency. Diagnosis of AADC deficiency was frequently delayed with almost half of the patients only receiving a definitive diagnosis more than 1 year after symptom onset; the average delay to diagnosis for the 16 patients reported in this case series was 3.2 years. For four older patients, a diagnosis was not achieved for many years. This is consistent with a large case series that demonstrated a longer latency from symptom onset to diagnosis in older vs younger children [17], perhaps reflecting an increasing awareness of this disease in recent years. Current guidelines recommend that a diagnosis of AADC deficiency should be based on the identification of compound heterozygous or homozygous pathogenic variants in the *DDC* gene combined with either CSF analysis or measurement of plasma AADC activity. In this study, AADC deficiency was genetically confirmed in all patients, although biochemical confirmation was not performed in six cases. These included three cases with variant c.1234C>T and one with c.1040G>C, classified as pathogenic, and one case with c.571-3C>6, reported as a warm variant of uncertain significance [20]. Two of the six cases had a family history of AADCd previously confirmed with both whole exome sequencing and plasma enzyme AADC activity assessment.

Brain MRI and EEGs were performed in the majority of cases, although findings were often normal or non-specific, indicating the limited use of these modalities in the diagnosis of AADC deficiency, other than to eliminate other more common conditions.

All patients in this case series had a homozygous variant within the *DDC* gene. This contrasts with other case series where compound heterozygous variants have been more common [17, 18]. This may reflect the fact that all patients in this series were born to consanguineous parents [21]. A novel intronic variant between exons 13 and 14 (c.1243–10 A>G) was identified (patient 2). Given that this was a novel variant, both plasma AADC enzyme activity and dried blood spot 3-OMD analysis were performed to confirm a AADC deficiency diagnosis. This patient had no tonus symptoms or motor impairment and was able to walk, talk, and had normal cognition. The patient presented with diarrhea and failure to thrive and has shown moderate improvements in response to treatment with vitamin B6. No patients in this case series had the intronic variant c.714+4A > T that is found frequently in patients from Taiwan or with Chinese ancestry; however, five patients had the c.1234C>T variant which has previously been reported in six Chinese patients and is associated with a severe phenotype [18]. Three patients had the intronic variant c.571-3C>G; this variant had previously been reported in a case from Saudi Arabia, although no clinical phenotype was described [22]. Other previously described variants include c. 206C>T and c. 242C>T that lead to amino acid substitutions in loop 1 [23, 24], c.175G > A that leads to a substitution in the N-terminal domain [18, 25], and c.1040G > A that leads to a substitution in loop 3 and is associated with a considerable decrease in catalytic activity [26].

Most patients received treatment with recommended first-line agents, including dopamine agonists, vitamin B6 and MAO inhibitors, along with a number of other drugs used to treat symptoms of the disease, e.g., anti-epileptics and anti-spasmodics. One patient responded well to treatment and at the time of writing is walking, talking, and attending school. This patient was diagnosed at a relatively early age (10 months) as he had an elder sibling already diagnosed with the condition. In contrast, his sibling was only diagnosed at 10 years of age and has not exhibited as good a response to treatment. In general, patients either showed no response to treatment or had only mild or moderate improvements in specific symptoms.

Five patients died prior to data collection with the cause of death attributed to the natural history of the disease. The average age at death was 7 years of age for the three patients with available data, which is in line with previously reported data. Premature death in patients with AADCd is common [3, 17, 27, 28], with the average age at death for Taiwanese patients with AADCd reported to be 4.6 years of age [28]. In a systematic review of case studies, 13 deaths were reported in a sample of 261 patients with AADCd [3]. Six of these patients died within 3 years of age. Bergkvist et al. reported an average age of death of 8 years of age based on 16 deaths in a sample of 185 [27].

This indicates a high unmet need for effective treatments for patients with AADC deficiency to treat both the underlying pathology and the symptoms. Gene therapy trials have demonstrated that delivery of a functional copy of the *DDC* gene using adeno-associated viral vector to the basal ganglia leads to clinical improvements [10, 13, 14]. Young age correlated with the clinical improvements, emphasizing the need for earlier diagnosis of AADC deficiency [13, 14]. The European Commission has recently granted marketing authorization for PTC Therapeutics’ product Upstaza™ (eladocagene exuparvovec), an in vivo gene therapy for treating AADC deficiency through delivery of the *DDC* gene to the putamen. It is approved for patients aged 18 months of age and older [29]. Therefore, raising awareness of AADC deficiency among primary care physicians in the Middle East region is key to ensure early access to future gene therapy treatments.

A strength of this study is that it highlights the heterogeneity in symptom and sign presentation for patients with AADC deficiency and the diagnostic challenges faced by healthcare professionals in the Middle East who manage patients with this rare condition. Limited AADC deficiency case reports have been reported from this region [17, 30–32]. Limitations are that it is based solely on patient cases and, as for all case reports, it is vulnerable to selection and recall bias. An incomplete dataset was captured for 2 patients; information on treatment dose and response is unreported.

The findings from this study confirm that delays in the diagnosis of AADC deficiency are common, even among patients who had no or limited attainment of motor milestones. This cohort of patients presented with a wide spectrum of signs and symptoms and did not always have typical symptoms, such as motor issues or oculogyric crises. Diagnostic follow-up was variable with all patients undergoing genetic testing, but secondary biochemical analyses were not always performed. AADC deficiency patients are frequently misdiagnosed with other conditions such as cerebral palsy, which can lead to delays in the initiation of treatments targeting the underlying pathology. In order to decrease the time to diagnosis and to reduce misdiagnoses, increased awareness of the condition is required. This could be achieved through introducing screening programs in at-risk patients for raised 3-OMD levels and the increased use of genetic testing using next-generation sequencing early in the diagnostic workup of patients. This may be particularly important in the Middle East region, where parental consanguinity and large family size can increase the prevalence of autosomal recessive genetic disorders. Increased awareness, research, and education are needed to guarantee timely diagnosis, management of AADC deficiency, and early access to future gene therapy treatments.

## Data Availability

The datasets generated during and/or analyzed during the current study are available from the corresponding author on reasonable request.
