# Correction to: Aromatic L-amino acid decarboxylase deficiency in countries in the Middle East: a case series and literature review

**DOI:** 10.1007/s00431-023-05012-1

**Published:** 2023-05-04

**Authors:** Musaad Abukhaled, Mohammed Al Muqbil, Malak Ali Alghamdi, Khalid Hundallah, Jehan Suleiman, Tawfeg Ben-Omran, Majid Alfadhel, Mohammed Almannai, Rehab Alsaleh, Brahim Tabarki

**Affiliations:** 1 Department of Neurosciences, King Faisal Specialist Hospital and Research Center, (KFSH-RC), Riyadh, Saudi Arabia; 2 College of Medicine, King Saud Bin Abdulaziz University for Health Sciences (KSAU-HS), Riyadh, Saudi Arabia; 3 Division of Pediatric Neurology, Department of Pediatrics, King Abdullah Specialized Children’s Hospital, National Guard Health Affairs (NGHA), Riyadh, Saudi Arabia; 4 King Abdullah International Medical Research Center, Ministry of National Guard Health Affairs (MNG-HA), Riyadh, Saudi Arabia; 5 Medical Genetic Division, PediatricDepartment, College of Medicine, King Saud University, Riyadh, Saudi Arabia; 6 Division of Neurology, Department of Pediatrics, Prince Sultan Military Medical City, Riyadh, Saudi Arabia; 7 Division of Neurology, Department of Pediatrics, Tawam Hospital, Al Ain, United Arab Emirates; 8 College of Medicine and Health Sciences, United Arab Emirates University, Al Ain, United Arab Emirates; 9 American Center for Psychiatry and Neurology, Abu Dhabi, United Arab Emirates; 10 Sidra Medicine and Research Center, Doha, Qatar; 11 Hamad Medical Corporation, Doha, Qatar; 12 Department of Genetics and Precision Medicine, Department of Pediatrics, King Saud Bin Abdulaziz University for Health Sciences, King Abdulaziz Medical City, Riyadh, Saudi Arabia; 13 Medical Genomic Research Department, King Abdullah International Medical Research Centre (KAIMRC), King Saud Bin Abdulaziz University for Health Sciences (KSAU-HS), Ministry of National Guard Health Affairs (MNG-HA), Riyadh, Saudi Arabia

**Correction to: European Journal of Pediatrics** **https://doi.org/10.1007/s00431-023-04886-5**

In the original published version of the above article, the *DDC* gene variant for patient 8 was incorrect in Fig. 4. This was given as c.245G > A; Homozygous; p.R82Q. This should be c.479G > A; Homozygous; p.R160Q. The text corresponding to Fig. 4 has also been corrected to “Six missense variants leading to a single amino acid substitution were reported for 12 patients across exons 2 (n = 1), 3 (n = 3), 5 (n = 1), 11 (n = 2) and 13 (n = 5).”

**Fig. 4:**
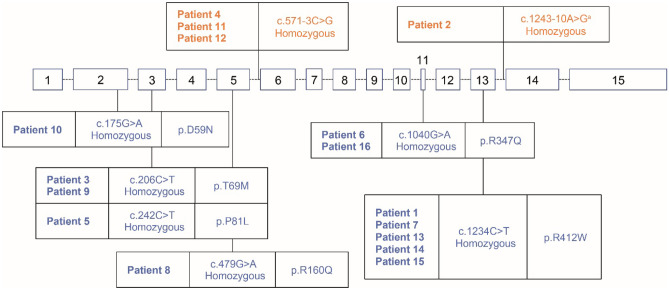
*DDC* gene variants. Genomic organization of the *DDC* gene and location of variants. Missense variants are indicated in blue and intron variants are indicated in orange. ^a^Previously unpublished variant. *DDC*, dopa decarboxylase

The original article has been corrected.

